# Healthcare Needs of an Older Adult Population Referred for Psychiatric Assessment in the Emergency Department of a University Hospital

**DOI:** 10.1192/bjo.2024.162

**Published:** 2024-08-01

**Authors:** Fiona Hoare, Ann O'Donoghue, Colm Sweeney, Darlene Tan, Meshari Alabdulrahman

**Affiliations:** 1 St Vincent's University Hospital, Dublin 4, Ireland; 2 St Patrick's University Hospital, Dublin, Ireland; 3 National Forensic Mental Health Service, Dublin, Ireland; 4 UCD, Dublin, Ireland

## Abstract

**Aims:**

It is estimated that approximately 15% of adults over 60 years old suffer from a mental disorder. Self-harm and suicidal ideation are associated with a range of mental disorders, and high rates of suicide. The aim of this project is to identify the characteristics of older people who present with self-harm and suicidal ideation to an emergency department (ED) in a university hospital. In examining the variables associated with self-harm we may be better able to identify the characteristics of older adults who are at highest risk.

**Methods:**

We conducted a cohort study of older adults (aged 65 years+) who presented to the Mater Misericordiae University Hospital with a mental health problem from 2008–2022 (a 15-year cohort). Data were extracted from the Electronic Patient Records including all patients who presented to the ED in that time period with a mental health triage code. We examined this cohort to collect detailed information on the characteristics of those older people presenting with self-harm and suicidal ideation.

**Results:**

We identified 30,941 ED attendances with a mental health triage code between 2008 and 2022. Of these, 946 (3.1%) were older adults. One-fifth (20%) presented with self-harm, a further 21% reported suicidal ideation. Of these, 8% reported previous self-harm and 32% had previously been reviewed by psychiatry. Over one-third (38%) were admitted. Of those, the majority (78%) were admitted to a medical or surgical ward, 16% to a psychiatric ward and 5% to critical care.

Of those presenting with self-harm 37% were admitted to hospital – 32% to a medical or other ward and 5% to psychiatric unit. There was a significant difference in those who were admitted with self-harm versus suicidal ideation (p < 0.001).

**Conclusion:**

Our results demonstrate key insights into older adults who presented to the ED with self-harm and suicidal ideation. These patients were more likely to be admitted to a medical ward than a psychiatric unit, and those with self-harm were more likely to be admitted medically compared with those with suicidal ideation.

Possible reasons for these results include the higher rate of medical co-morbidity in older adults and the potential high lethality of self-harm in this cohort. Another explanation could be the scarcity of acute psychiatric beds necessitating medical admission. There is a need for further exploration of this high-risk population.

